# Synchronous Vibration Measurements for Shrouded Blades Based on Fiber Optical Sensors with Lenses in a Steam Turbine

**DOI:** 10.3390/s19112501

**Published:** 2019-05-31

**Authors:** Dechao Ye, Fajie Duan, Jiajia Jiang, Zhonghai Cheng, Guangyue Niu, Peng Shan, Jiamin Zhang

**Affiliations:** 1 State Key Laboratory of Precision Measuring Technology and Instruments; Tianjin University, Tianjin 300072, China; fjduan@tju.edu.cn (F.D.); jiajiajiang@tju.edu.cn (J.J.); chzhh@tju.edu.cn (Z.C.); niuguangyue@tju.edu.cn (G.N.); 2 Shanghai Electric Power Generation Equipment Co., Ltd. Turbine Plant, Shanghai 201111, China; shanpeng@shanghai-electric.com (P.S.); zhangjm10@shanghai-electric.com (J.Z.)

**Keywords:** blade vibration measurement, blade tip timing, shrouded blades, steam turbine, fiber optical sensor, nodal diameter

## Abstract

It is important to obtain accurate dynamic vibrations of steam turbine blades for safe operation. Strain gauge (SG) measurements cannot fully obtain vibrations of all blades and nodal diameter (ND) details. The blade tip timing (BTT) technique could resolve this problem because it has the advantage of measuring all blades’ vibrations. This article proposed an improved BTT technique for measuring synchronous vibrations of shrouded blades in a steam turbine based on fiber optical sensors with lenses. The newly developed sensor was equipped with a Plano-convex lens, and the optical path was specifically designed to collimate the beam within a large working distance from 4 to 19 mm and improve the measuring accuracy. A method to search the spectra of all peak vibration amplitudes of all the blades was proposed to obtain the ND details of synchronous vibration. Experimental results validated the efficiency and accuracy of the proposed methods and sensors. Comparison results of BTT measurements with SG measurements showed that the relative errors of normalized frequency and strain were small and acceptable.

## 1. Introduction

With the development of power plants towards high power and intelligent operation, vibrations or failures of blades, especially the last stage blades in steam turbines, have happened more frequently as a result of unstable operating conditions brought by flexible operation. It is important to obtain accurate dynamic characteristics of steam turbine blades for safe operation [1,2]. A telemetry system has been used to measure the strain of selected blades in a steam turbine, but the installation and maintenance of strain gauge measuring systems are complicated and expensive [3]. Besides, it cannot obtain the maximum strain if the SG is not placed at that blade. The ND details could not be obtained without vibration information of all blades.

The blade tip timing (BTT) technique has been used with considerable success for blade vibration measurements, where several optical or other types of probes are mounted on the machinery casing to measure blade tip arrival times. In the presence of vibration, each blade passes the probe at an earlier or later time compared to nonvibration cases [4,5,6,7,8,9]. By further processing those arrival times, the vibration of all blades in the circumferential direction can be obtained [10,11,12,13,14,15,16,17,18]. The noncontact BTT method could monitor all blade vibration frequencies and deflections. It is useful for dynamic characteristic measurements of a bladed disk in a steam turbine. Kubín at the University of West Bohemia in Pilsen, performed BTT measurements in power stations and investigated the calibration of tip timing sensors using passive eddy current sensors for shrouded blades. An optical sensor from HoodTech Corporation was also used in the test rig as reference for a quick overview of exiting [19]. The best sensor axial position was calculated and verified by the FEM model and experiment. The HoodTech Corporation proposed an opposite type of fiber optical tip timing sensor with a lens to sense the arrival times of the side edge of shrouded blades. It was difficult to install the receiving terminal of the sensor because the receiving terminal should be inserted into the flow passage of the rotor [20].

Together with the Shanghai Electric Power Generation Equipment Co., Ltd., the authors also attempted to use BTT technology instead of the telemetry method to measure the vibration parameters of a newly designed blade with an interlocked shroud, and they validated tip timing measurements using fiber optical timing sensors without lenses [21]. This paper developed a new fiber optical sensor with a lens to improve the accuracy of blade vibration measurements. Based on improvements of the signal to noise ratio (SNR) of the blade’s arriving signal, a method to obtain the ND details of synchronous vibrations was proposed. This paper mainly focused on application of the BTT technique in synchronous vibration measurements for shrouded blades of a steam turbine based on fiber optical sensors with lenses. The main contents of the article are listed as follows:

1. An improved BTT technique that utilized the character of an interlocked shroud structure was proposed to sense the circumferential displacements caused by bladed disk vibrations in the axial direction.
2. A type of fiber optical sensor with a Plano-convex lens was developed, which collimated the beam and kept the measuring spot diameter less than 1.20 mm within a large working distance from 4 to 19 mm. A special optical path in the sensor head was designed to improve the signal to noise ratio (SNR) of the blade’s arriving signal, which assured a high accuracy of blade vibration measurements.
3. The least squares fitting method was suggested to identify synchronous vibrations of the shrouded blades. This paper provided a spectrum peak searching method for obtaining the ND details of synchronous vibrations.
4. Vibration tests for the last stage blades of a steam turbine were carried out in a high-speed dynamic balance laboratory, and SG measurement results of two blades were obtained simultaneously for comparison. Analysis results validated the efficiency and accuracy of the proposed methods and sensors.

## 2. Methodology

### 2.1. An Improved Blade Tip Timing (BTT) Technique for Shrouded Blades

**Figure 1:**
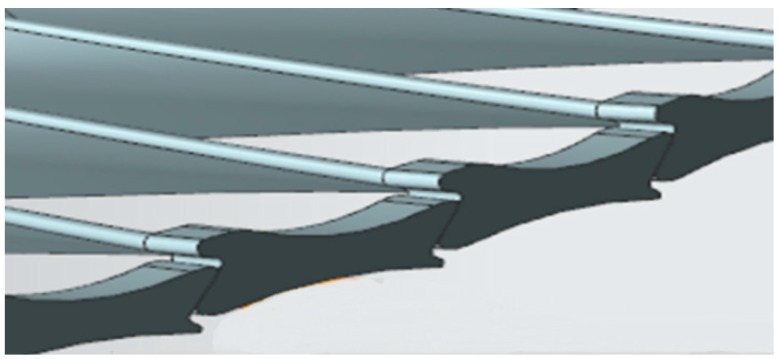
Shrouded blades of the last stage in a steam turbine.

A rotor with shrouded blades in a steam turbine is a typical structure for a bladed disk. Shrouds are introduced to increase stiffness and also provide interblade coupling, as shown in Figure 1. Since a bladed disk is a cyclically periodic structure, it exhibits vibration modes where the mode shape has nodal diameters and the direction of vibration is axial. The bladed disk’s vibration mode can be extracted using nonlinear finite element analysis tools to evaluate the dynamic vibration performance [22,23]. It seemed that no vibration in the rotational direction was produced in the bladed disk, and certain modifications and improvements based on BTT techniques should be made for shrouded blades of a steam turbine.

**Figure 2:**
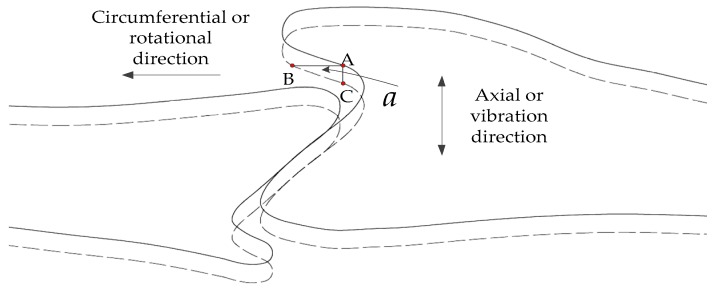
The interlocked shroud structure of a steam turbine.

The interlocked shroud structure of steam turbine blades is shown in Figure 2. The edge between point B and C is nearly a straight line, and the angle between line BC and rotation direction is *a*. In this paper, this character of the interlocked shroud structure was utilized for measuring blade vibrations based on the BTT technique.

Assuming the measuring spot (or the so-called sensing area) is among points B and C, the circumferential displacement would be produced when the blade vibrates in the axial direction. Then, the circumferential displacement of all blades could be obtained by the BTT method. The actual vibration deflection from point A to point C would lead the measuring point to change from point A to point B. The relationship between actual vibration amplitude (*A_actual_*) and the amplitude result of BTT measurements (*A_BTT_*) is linear and described as follows:

(1) $$A_{actual}=A_{BTT}\times \tan(a).$$

However, *a* at a certain rotational speed would be changed because of the centrifugal force. The value of *a* at a certain rotational speed can be obtained from the designer using the FEM analysis method, which was used in this paper. Another more accurate method was suggested using two fiber optical tip timing sensors. The two sensors were mounted at two different axial positions on line BC. Then, the actual values of *a* could be measured in real time. In Figure 2, the length of line AB (*L_AB_*) and AC (*L_AC_*) are about 5 and 2 mm, respectively. The measuring spot should be as small as possible. A large measuring spot will lead to failure to use the character of the interlocked shroud structure, and Equation (1) cannot be used to describe the relationship between *A_actual_* and *A_BTT_*. It is noteworthy that *L_AC_* would be the maximum value of vibration displacements for ensuring compliance with Equation (1).

When a blade tip passes through the measuring spot, the rising time of the signal ($T_{r}$) is proportional to the diameter of the measuring spot ($d_{s}$). Assuming the velocity of the blade tip is v in the circumferential direction, $T_{r}$ can be described as follows:

(2) $$T_{r}=\frac{d_{s}}{v\times \sin(a)}.$$

Tip timing error (δt) is also proportionally related to $T_{r}$ [24]. Assuming $\delta t=\lambda \times T_{r}$, λ is related to the SNR of tip timing sensors and the signal condition in the BTT system. Generally, λ could be less than 0.1 with current developments of BTT techniques. Assuming the measurement error of *A_BTT_* is δd, and δd=δt×v, the relative measuring error can be derived from Equation (3).

(3) $$\delta d/L_{AB}=\lambda \times d_{s}/(L_{AB}\times \sin(a)).$$

In Equation (3), the relative measuring error will be 6.697% when $d_{s}$ = 1.2 mm and *a* = 21°. The diameter of the measuring spot should be as small as possible to utilize the interlocked shroud structure’s character of steam turbine blades.

### 2.2. A Fiber Optical Sensor with a Lens

**Figure 3:**
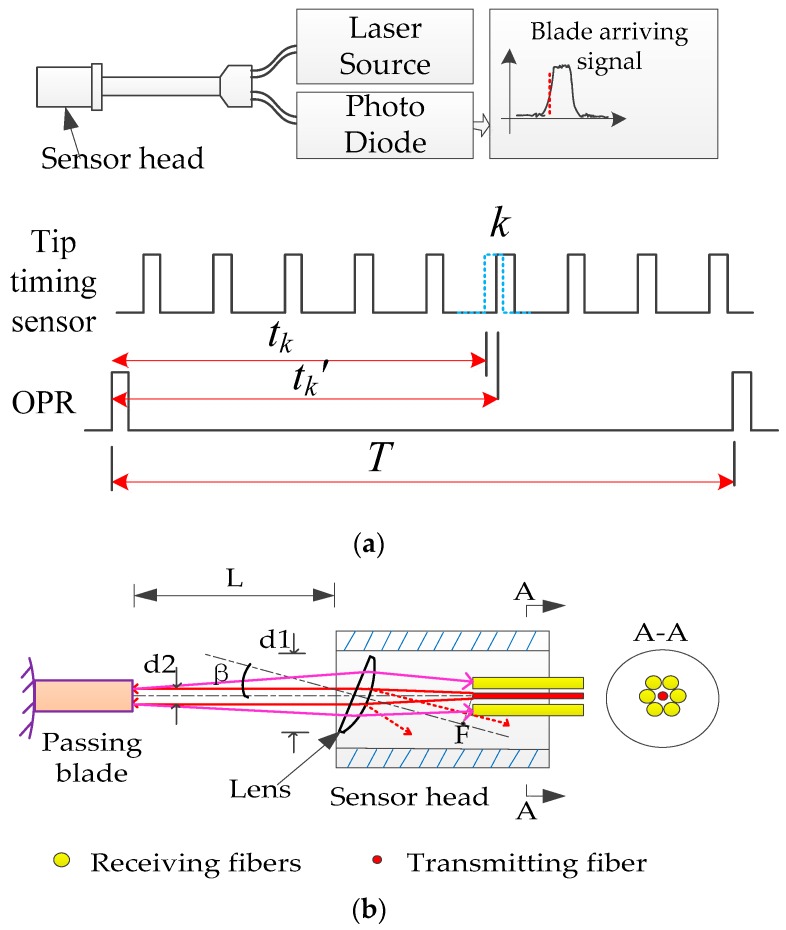
(**a**) The typical fiber optical tip timing sensor. (**b**) The sensor head of the fiber optical sensor with a lens.

According to the above description, the measuring spot of a tip timing sensor should be small enough within a large range of working distance. The working distance range might change from 5 to 18 mm in different types of steam turbines. An innovative fiber optical sensor with a lens was proposed to satisfy all of the requirements of the tip timing sensor. The proposed fiber optical sensor with a lens was similar to a typical fiber optical tip timing sensor, which consists of a transmitting fiber in the center of the sensor head and six receiving fibers around, as shown in Figure 3a. The sensor head is mounted on the engine casing and transmits a beam of light from a laser source. A photo diode converts the reflected light into a voltage output when a blade passes through. The voltage is related to the intensity of reflected light, which would vary with the angle of the blade tip surface and the distance between the sensor head and blade tip. However, work of tip timing sensor is based sensing all the arrival times of “passing blades”, which are the occurrences of reflected light. It is not sensitive to the intensity of reflected light. A small intensity of reflected light would affect the SNR of the blade’s arrival signal, so the sensor is equipped with six receiving fibers. Figure 3a shows the basic principle of tip timing. Generally, the BTT system consists of four or more tip timing sensors, a once per revolution (OPR) sensor, signal conditioners, timing card, and BTT data analysis software. The blade arriving signal would be conditioned into pulses. The times of rising or falling edges are recorded by the timing card. If a blade was vibrating, the pulse would occur in an earlier or later time compared to the nonvibration stage. Then, data analysis software could obtain tip displacements in the circumferential direction and restore the vibration amplitude and frequency using a well-established model fitting method. Assuming the diameter of a rotating blade tip is *D*, the formula to calculate the displacement of blade *k* in Figure 3a could be described as:

(4) $$y_{k}=\pi D/T\times (t_{k}-{t_{k}}^{\prime }).$$

In a typical fiber optical tip timing sensor, the diameter of the beam will become larger when the working distance increases. In addition, the intensity of the arriving signal will become rapidly smaller, and the optimal working distance is less than 5 mm. In order to solve these problems, a lens was introduced in front of the fibers, as shown in Figure 3b.

**Figure 4:**
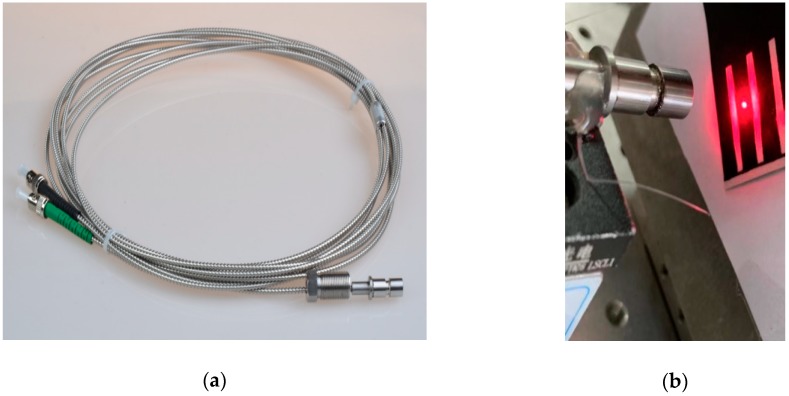
(**a**) Photograph of a fiber optical sensor with a lens. (**b**) Photograph of the measuring spot when the working distance is 19 mm.

Angle β was specially designed between the optical path and the lens, and it was a Plano-convex lens. β should be set at an appropriate value to make sure that reflected light from the lens would not solely be sensed by receiving fibers to improve the SNR of the blade’s arriving signal. This was very useful because the intensity of reflected light from surface of the lens might be larger than that from passing blades, especially when the working distance was about up to 8 mm. It was noteworthy that angle β should also be set small enough, because it changed the angle of reflected light from the blade, which was finally received by the receiving fibers. A bigger value of β would lead to a slight decline in voltage. When the blade was rotating, the angle between the reflection surface and the optical path changed as well. The voltage would reach a peak while the specular reflection of light was received. The transmitting fiber should be located at the focal plane of the lens, and then the beam of a transmitting fiber would be collimated. Assuming the transmitting fiber’s core diameter was 200 μm and the numerical aperture (NA) was 0.22, the diameter of collimated beam (d2) will be less than 1.2 mm when the focal length of the lens is 4 mm. Another important contribution using a Plano-convex lens is that it will increase the reflected light’s receiving aperture compared to receiving signal directly by fibers. Hence, the working distance (*L*) could be enlarged to 18 mm while the diameter of the measuring spot was still kept to be less than 1.2 mm. The photograph of a fiber optical sensor with a lens is shown in Figure 4.

**Figure 5:**
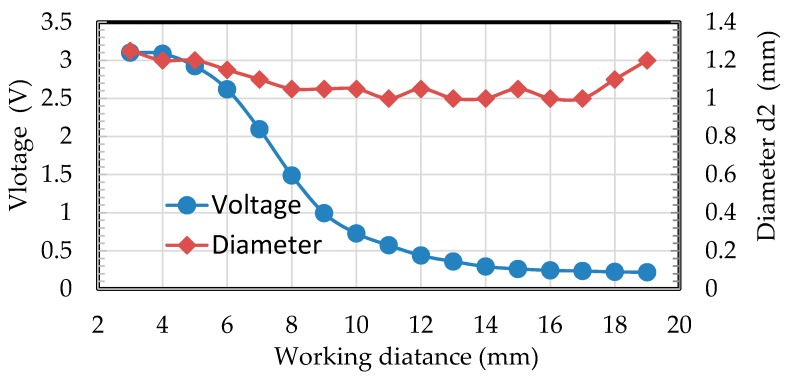
Results of the sensor’s performance.

Figure 5 shows that the beam of the transmitting fiber was collimated successfully. The diameter of the measuring spot was kept in the range from 1.0 to 1.25 mm. Although the voltage conversed from the reflecting light became smaller during a distance range from 8 to 19 mm (0.2 V in the distance of 19 mm), the noise of the voltage was under 0.01 V and the SNR was up to 20. The diameter of the measuring spot from the same fiber optical tip timing sensor without a lens was larger than 3.5 mm when the working distance was 15 mm. According to Equation (3), the relative measuring error of traditional optical sensors would be 19.533%.

### 2.3. Identification of Synchronous Vibration and Nodal Diameter (ND) Details

When synchronous vibration with the nodal diameter occurs in steam turbine blades, the vibration frequency is an integral multiple of rotational frequency. The integral number is also called an engine order (EO). The blade tips will have the same displacements every time they pass the sensor. Therefore, analysis of BTT data to identify synchronous vibration becomes a lot more difficult. The least squares fitting method, also well known as the circumferential Fourier fitting method, is usually used to identify synchronous vibrations for free blades of rotational machinery [25]. It can also be used for shrouded blades of steam blades, which is briefly described as follows.

Assuming the vibration displacement measured by Sensor *i* located at azimuth *α_i_* from the reference sensor is:

(5) $$y_{i}=A\sin(N_{e}\alpha_{i}+\phi )+C,$$

where *A* and *φ* are the amplitude and phase of vibration; $N_{e}$ refers to EO; *i* is the sensor number; and *C* is the blade deflection caused by centrifugal loading, twist, shaft torsion, etc.

Regrouping the measurements of $y_{i}$ into a vector Y, the parameters *A*, *φ*, and *C* satisfy the following equation:

(6) Y=BX,

where

(7) $$Y={\left(\begin{array}{ccccc} y_{0} & y_{1} & y_{2} & \cdots & y_{n-1} \end{array}\right)}^{T},$$

(8) $$X=\left[\begin{array}{l} x_{1}\\ x_{2}\\ x_{3} \end{array}\right]=\left[\begin{array}{l} A\sin\phi \\ A\cos\phi \\ C \end{array}\right],$$

(9) $$B=\left[\begin{array}{ccc} \cos(N_{e}\alpha_{0}) & \sin(N_{e}\alpha_{0}) & 1\\ \cos(N_{e}\alpha_{1}) & \sin(N_{e}\alpha_{1}) & 1\\ \cos(N_{e}\alpha_{2}) & \sin(N_{e}\alpha_{3}) & 1\\ \vdots & \vdots & \vdots \\ \cos(N_{e}\alpha_{n-1}) & \sin(N_{e}\alpha_{n-1}) & 1 \end{array}\right].$$

If *N_e_* is known, the other parameters could be obtained using the least squares fitting method. The parameter vector $X_{fit}$ is given by the following formula:

(10) $$X_{fit}={\left(B^{T}B\right)}^{-1}B^{T}Y.$$

The residual is:

(11) $$E_{fit}=BX_{fit}-Y,$$

where $E_{fit}={\left(\begin{array}{ccccc} e_{0} & e_{1} & e_{2} & \cdots & e_{n-1} \end{array}\right)}^{T}$. The root mean square of $E_{fit}$ (σ) is given by the following formula:

(12) $$\sigma =\sqrt{\frac{\sum_{i=0}^{n-1}e_{i}^{2}}{n}}.$$

σ provides a confidence index for the fit, and the best $N_{e}$ is obtained when σ is a minimal value.

In order to obtain the ND details once the amplitudes of all blades have been already identified, a spectrum peak searching method was proposed as follows:

1. Set the sampling rate equal to the total number of blades (*N_b_*), and then make sure that all blade amplitudes are arranged in a sequence.
2. Perform an *N_b_*-point FFT operation and search the peak in that FFT spectrum. The peak will be located at the position of 2*k* Hz if the nodal diameter is *k*.

## 3. Experiments, Results, and Discussion

### 3.1. Experiment Setup

**Figure 6:**
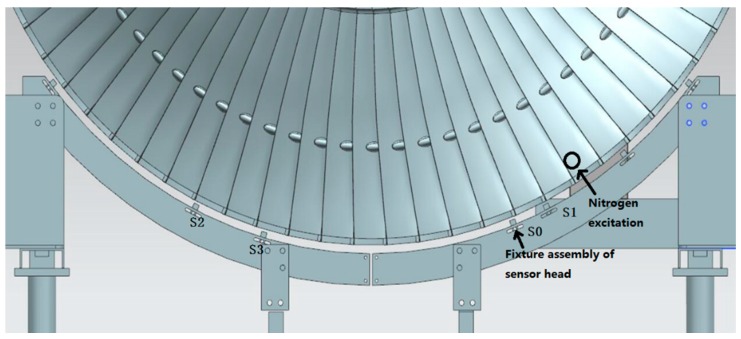
Locations of sensors, nitrogen excitation, and strain gauges.

**Figure 7:**
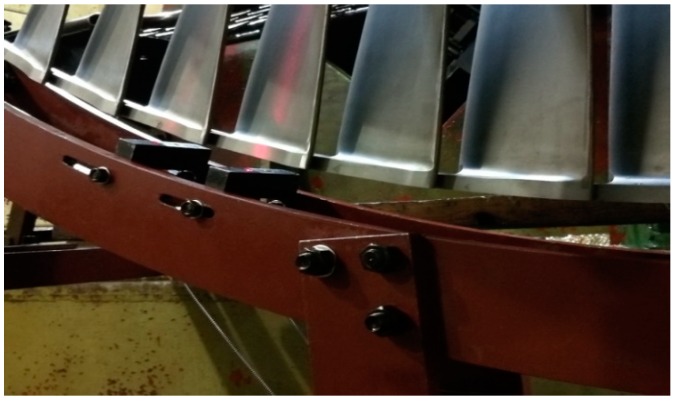
Installations of fiber optical sensors.

In order to validate the effectiveness of the improved BTT method for shrouded blades based on the fiber optical sensor with a lens, vibration tests for the last stage blades of a steam turbine were carried out in a high-speed dynamic balance laboratory. Four fiber optical sensors with lenses were installed at the fixture assembly of sensor heads, which were labeled as S0, S1, S2, and S3. The locations of measuring spots were adjusted at points B and C at the interlocked shroud structure of the shrouded blades. The working distance of the sensor was set up to 12 mm for safety considerations. The rotational speed was controlled as it decreased from 3300 to 2000 rpm in the tests, and a nitrogen excitation system was used to excite the resonant vibrations. Synchronous vibrations will occur when the vibration frequency is an integral multiple of rotational frequency and the EO is equal to ND [21]. For comparison with strain gauge method, a telemetry system for sensing the changes of strain was used during the tests. Two strain gauges were placed at the root of the blades. The locations of the strain gauge were derived from FEM predictions of strain distribution. The locations of sensors, strain gauges, and nitrogen excitation are shown in Figure 6, and the installations of sensors are shown Figure 7.

**Figure 8:**
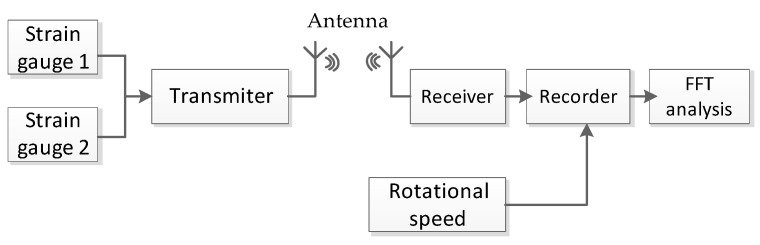
Block scheme of the telemetry system for strain gauge tests.

In the telemetry system, the transmitter modulated the strain signals and sent them to the receiver by antenna, shown in Figure 8. The recorder recorded the test data, and then the blade vibration response and vibration spectrum were processed using FFT analysis tools. Thus, vibration information including frequency and amplitude were all obtained.

### 3.2. Analysis Results of Synchronous Vibrations

#### 3.2.1. Analysis of Vibration Amplitudes

**Figure 9:**
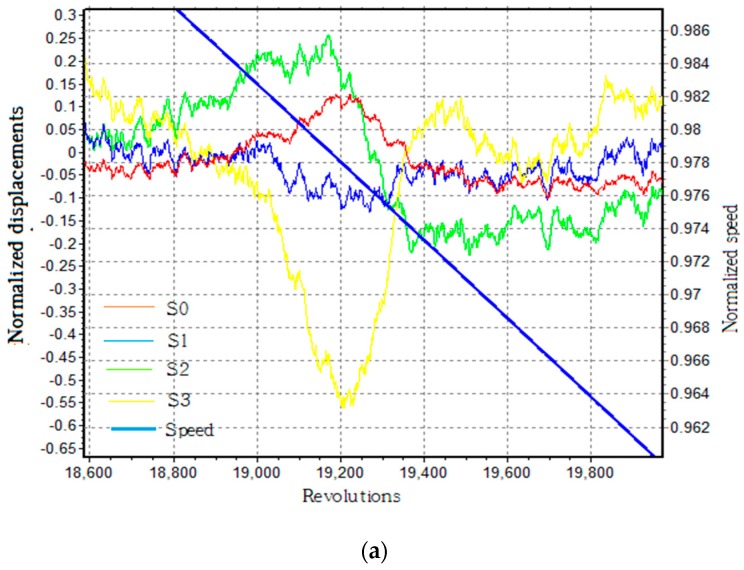

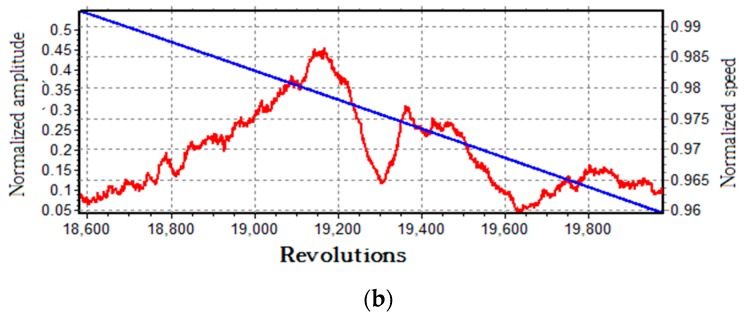
(**a**) Normalized displacements of all sensors (blade 22#). (**b**) Normalized amplitude results using the least squares fitting method.

During the decline of rotational speed, synchronous vibration occurred when the vibration frequency was an integral multiple of rotational frequency with the help of the nitrogen excitation system. Figure 9a shows that all fiber optical sensors sensed the changes of displacements in blade 22# when the normalized speed was about 0.978, (based on the displacement waveforms of sensors S0, S1, S2, and S3). In this paper, results were normalized by means of dividing a constant value to keep the commercial and technical secrets of turbine plant strictly confidential. All the actual speeds results were divided by a constant value of A1 directly, all vibration displacements and amplitudes were divided by a constant value of A2, and the strain results were divided by a constant value of A3. Thus, important information including trends and interrelations of results are completely reserved. The least squares fitting method was used to fit the BTT data, and results are shown in Figure 9b. The normalized amplitude reached a maximum of 0.467 at the speed of 0.9788, and the best fit of EO was 5. In the tests, synchronous vibrations also occurred at 1.2592 when EO was 4.

**Figure 10:**
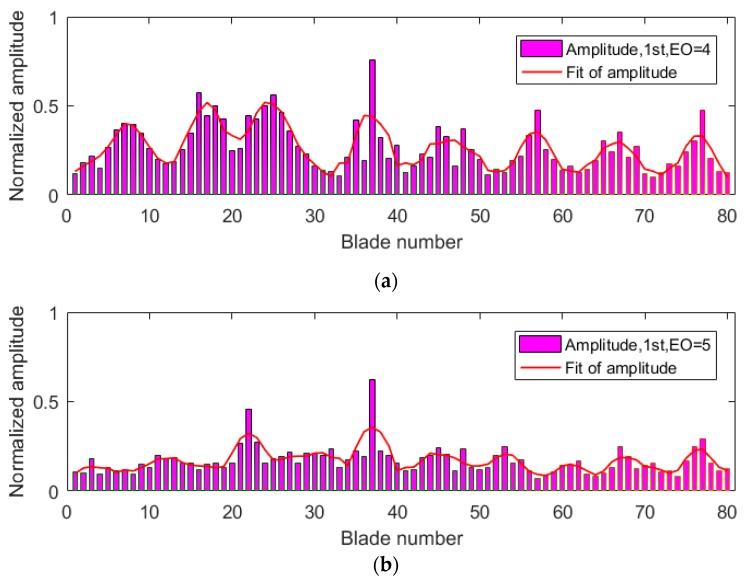
Normalized amplitudes of all blades in the first test: (**a**) engine order (EO) = 4 and (**b**) EO = 5.

**Figure 11:**
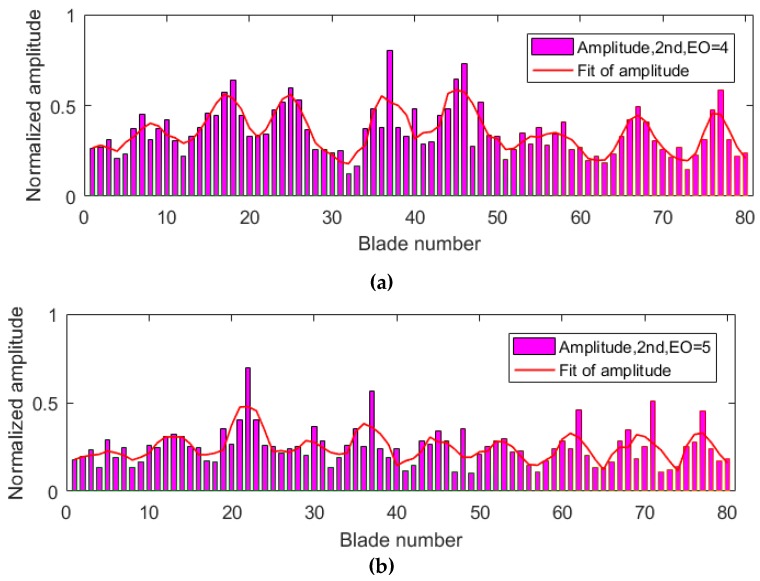
Normalized amplitudes of all blades in the second test: (**a**) EO = 4 and (**b**) EO = 5.

The same tests were carried out twice to validate the efficiency and accuracy of the proposed methods in the paper. The normalized amplitude results of two tests are shown in Figure 10 and Figure 11. Synchronous vibrations both occurred when EO = 4 and EO = 5 during the two tests. Since a larger force of nitrogen was excited in the second test, the average amplitude of all blades in the second test was larger than in the first test. Figure 10 and Figure 11 also indicated that the amplitudes varied with changes of blade numbers, which were more obvious using a polynomial fitting. Theoretically, shrouded blades of steam blades are cyclically periodic structures, and the synchronous vibration mode shape has an ND. The number of periodic changes in amplitude will be twice that of ND. Experimental results in Figure 10 and Figure 11 verified that the ND in synchronous vibrations of shrouded blades was actually equal to EO. Successful measurements of ND results also verified the accuracy of the proposed sensor with a lens, as it had smaller errors and could distinguish periodic variations of amplitudes.

#### 3.2.2. Analysis of ND Details

**Figure 12:**
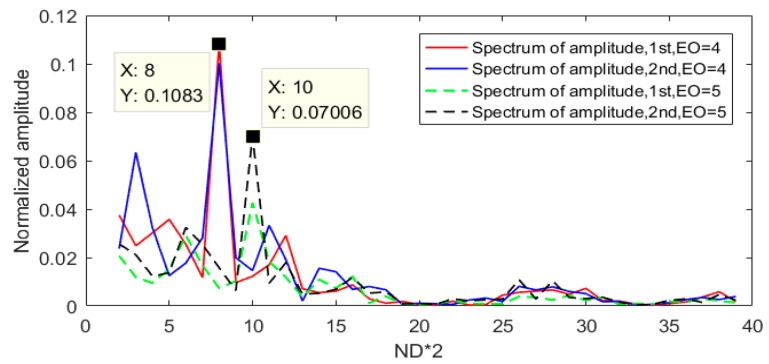
FFT spectra of all blade amplitudes.

In order to obtain more details about ND in synchronous vibrations, a spectrum peak searching method was used, and the results are shown in Figure 12. Four spectrums of all blade amplitudes reached peaks at 2*k* Hz (*k* refers to ND).

**Figure 13:**
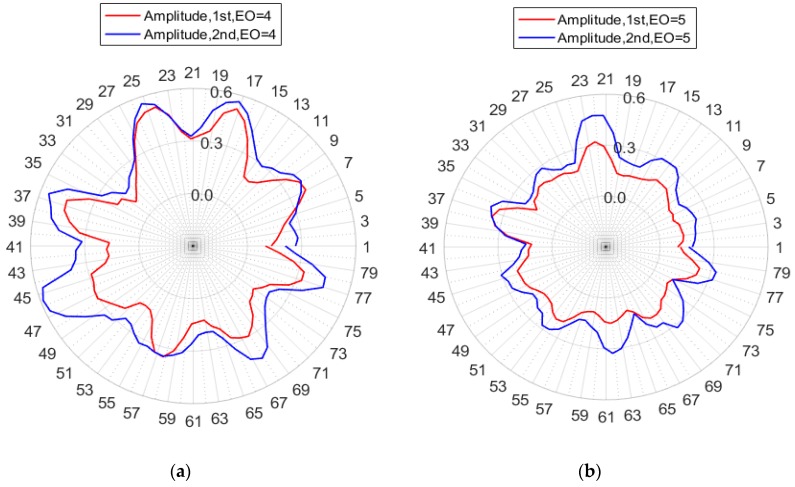
Polar plots of all blade amplitudes: (**a**) EO = 4 and (**b**) EO = 5.

Polar plots might provide a more compressive and clear display of ND details for synchronous vibrations in shrouded blades, as shown in Figure 13. All amplitudes vs. blade numbers were plotted in a circular graph, and the results of the two tests were plotted in the same figure. Figure 13a clearly showed that the ND was 4 and the four nodal lines were located at blade numbers 1-41, 12-52, 21-61(or 62), and 32-72(or 73). The locations of nodal lines were almost identical in two tests, indicating that the nodal lines of synchronous vibrations were constant in the tests. The SG measuring system might not get the maximum vibration amplitude in synchronous vibrations of shrouded blades if the SG was not placed at that blade.

The same conclusion could be derived from Figure 13b, despite that the amplitudes were smaller than EO = 4. Results of Figure 12 and Figure 13 clearly validated the efficiency of the proposed methods in this article. The methods could obtain both the amplitudes of all blades and the ND details of the bladed disk.

#### 3.2.3. Analysis of Vibration Frequencies

**Figure 14:**
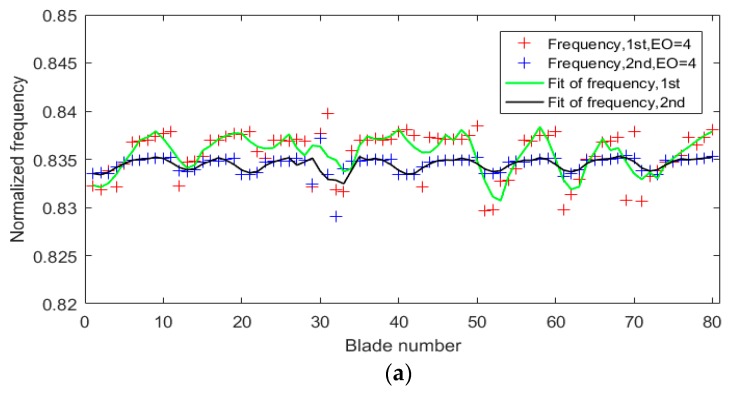

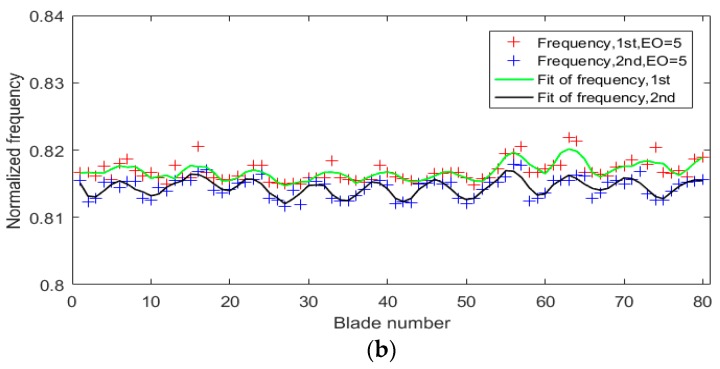
Comparison of normalized frequencies: (**a**) EO = 4 and (**b**) EO = 5.

Because of the inconsistency among blades, the amplitudes of different blades might reach peaks at different resonant speeds, meaning that vibration frequency measurements were a little different. Figure 14 shows the normalized frequency results of all blades. The temperature of the blades was slightly higher in the second test than the first test during the continuous experiments. The averaged normalized frequency of the second test was smaller than the first test, which was validated in Figure 14.

**Figure 15:**
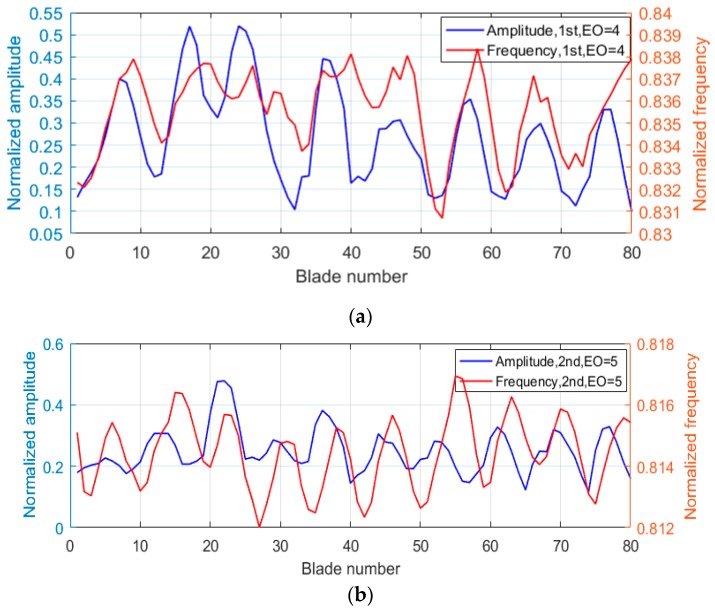
Comparison of normalized frequencies with amplitudes: (**a**) first, EO = 4 and (**b**) second, EO = 5.

Figure 14 indicated that the normalized frequencies varied with blade numbers using a polynomial fitting. The number of periodic changes in the normalized frequencies of all blades was twice that of EO or ND. This could be explained by the degree of intershroud coupling or by the differences in stiffness between each other when synchronous vibration occurred. Generally, the frequency would be greater with the increase of intershroud coupling and stiffness, which are related to the vibration amplitude. Therefore, the number of periodic changes in normalized frequencies of all blades was also twice that of EO. Comparison results of normalized frequencies with normalized amplitudes are shown in Figure 15, which verified the above explanation. Figure 15a shows the comparison results in the first test when EO = 4, and Figure 15b shows the second test when EO = 5. The changes in frequencies were slightly slower than in amplitudes, indicating that the maximum changes of stiffness were not exactly located at the points of maximum amplitude.

### 3.3. Comparisons of BTT Measurements with Strain Gauge (SG) Measurements

Two strain gauges were placed at blade 11# and blade 52#, and the strains were measured simultaneously during the second test. Comparison results of BTT measurements with SG measurements are shown in Table 1. The normalized stains from BTT measurements were calculated using the ratio (*R_FEM_*) between the strain at the point of strain gauge and the blade tip’s circumference amplitude. *R_FEM_* was obtained from finite element simulation data at the resonant mode, and then the normalized amplitudes were multiplied by *R_FEM_*.

From the results in Table 1, the relative errors of normalized frequency between BTT measurements and SG measurements were less than 0.348%. The results of BTT measurements agreed well with SG measurements, which approved the efficiency and accuracy of the proposed method in this paper. Despite that the relative error of normalized strain in blade 11# when EO = 5 was up to 23.433%, the average error was less than 10% within the engineering permissible range. This proved that the accuracy of BTT measurements in shrouded blades using fiber optical sensors with lenses was similar to those applications in free blades. There are many factors that influence the comparison results of blade 11# when EO = 5, such as the error of the measuring spot’s location and the signal quality of BTT data, which would introduce measuring error in the circumference displacements. The error of *R_FEM_* and the error of SG measurements would also affect comparison results.

## 4. Conclusions

This paper mainly presents synchronous vibration measurements for shrouded blades in a steam turbine based on fiber optical sensors with lenses. An improved BTT technique for shrouded blades utilizing an interlocked shroud structure was proposed to sense circumferential displacements caused by bladed disk vibrations in the axial direction. A kind of fiber optical sensor with a Plano-convex lens was developed, which collimated the beam and kept the diameter of the measuring spot less than 1.20 mm within a large working distance from 4 to 19 mm. A special optical path in the sensor head was designed to improve the SNR of the blade’s arriving signal, which assured a high measuring accuracy of the method. The least squares fitting method was suggested to identify synchronous vibrations of shrouded blades. Then, this paper provided a spectrum peak searching method for obtaining ND details of synchronous vibration. Vibration tests for last stage blades of a steam turbine were carried out in a high-speed dynamic balance laboratory to validate the efficiency and accuracy of the proposed methods and sensors.

Experimental results showed that the synchronous vibrations of all blades were successfully detected using fiber optical sensors with lenses, and the ND details were also obtained from BTT measurements, which could not be detected by SG measurements. The nodal lines were positioned in the polar plots. From analysis results of vibration frequencies, the sensed frequencies of all blades also indicated that the bladed disk vibration modes were full with interblade coupling. A comparison of results of BTT measurements with SG measurements in the experiments showed that the relative error of normalized frequency was less than 0.348%, and the average relative error of normalized strain was less than 10%. Through these experimental results, we are confident that the BTT technique can be applied to vibration measurements of shrouded blades in steam turbines using fiber optical sensors with lenses.

Although only synchronous vibrations were excited in the experiments, other types of blade vibrations, which could lead to a change of circumferential displacements, can also be measured using the method provided in this paper. Thus, different BTT data analysis methods for other types of vibrations in steam turbines can be further studied.

## Figures and Tables

**Table 1:** Comparison results of blade tip timing (BTT) measurements with SG measurements.

Items of Comparison	EO	Blade Number	SG Measurements	BTT Measurements	Relative Errors
Normalized frequency	4	11	0.8395	0.8378	−0.203%
Normalized frequency	5	11	0.8157	0.8159	0.025%
Normalized frequency	4	52	0.8327	0.8298	−0.348%
Normalized frequency	5	52	0.8159	0.8158	−0.012%
Normalized strain	4	11	0.9151	0.8520	−6.895%
Normalized strain	5	11	0.6653	0.5094	−23.433%
Normalized strain	4	52	0.7301	0.7169	−1.808%
Normalized strain	5	52	0.5502	0.5752	4.544%
